# Recycled Aggregates Influence on the Mechanical Properties of Cement Lime-Based Mortars

**DOI:** 10.3390/ma17205122

**Published:** 2024-10-21

**Authors:** Saitis Catalin, Manea Lucia Daniela, Marioara Moldovan, Plesa Luminita Monica, Gheorghe Borodi, Ioan Petean, Letiu Sorin

**Affiliations:** 1 Faculty of Civil Engineering, Technical University of Cluj-Napoca, 28 Memorandumului, 400114 Cluj-Napoca, Romania; daniela.manea@ccm.utcluj.ro (M.L.D.); sorinletiu@yahoo.com (L.S.); 2 Department of Polymer Composites, Raluca Ripan Institute for Research in Chemistry, Babeș-Bolyai University, 30 Fantanele Street, 400294 Cluj-Napoca, Romania; marioara.moldovan@ubbcluj.ro; 3 National Institute for Research and Development of Isotopic and Molecular Technologies, 65-103 Donath Street, 400293 Cluj-Napoca, Romania; borodi@itim-cj.ro; 4 Faculty of Chemistry and Chemical Engineering, Babeș-Bolyai University, 11 Arany Janos Street, 400028 Cluj-Napoca, Romania; petean.ioan@gmail.com

**Keywords:** plastering mortars, sustainable development, construction waste, mineralogical investigation, fractography

## Abstract

The current framework for managing construction waste, guided by European Union regulations, calls for an integrated waste management system. However, the reuse of old plaster waste, particularly from deteriorated facades, remains underexplored. This study investigates the potential of repurposing old plaster waste as a substitute for aggregates and cement in mortars, with the aim of promoting environmental sustainability and resource efficiency. Three mortar mixes were analyzed: a control mix, a mix with 45% waste replacing aggregates, and a mix with 10% waste replacing cement. Results show that replacing 45% of aggregates with plaster waste led to a 30% reduction in flexural strength, while the 10% cement replacement increased flexural strength by 6%. Compressive strength dropped by 27% and 38% for cement and aggregate replacements, respectively. Despite these reductions, the waste replacement remained within acceptable limits for structural integrity. Further microscopic analysis revealed that the incomplete integration of portlandite particles from the waste contributed to non-uniform bonding and crystal formation, weakening the mortar’s structure. This research demonstrates the feasibility of reusing old plaster waste, offering a novel approach to reducing construction waste and promoting a circular economy. It contributes to filling the knowledge gap on the reuse of plaster mortars while aligning with sustainable construction goals.

## 1. Introduction

The need for sustainable development became apparent in the 1970s due to rising concerns about pollution and environmental degradation. This period saw the creation of strategies to reduce the negative effects of economic growth on the planet’s resources. The construction industry is a major contributor to global waste, resource use, and carbon emissions, accounting for 40%. As a result, research has focused on recycling construction waste by incorporating it into building materials. Continuing such research is vital for future sustainability efforts [1,2].

Various materials like fly ash, slag, recycled concrete, rubber, glass, ceramic waste, and agricultural residues have shown potential for reuse in construction, either as aggregate replacements or partial cement substitutes. These practices are key to reducing the industry’s environmental impact [3].

One pressing challenge is the waste from plaster mortars in aging buildings, especially as facade restorations increase. This waste can make up to 100% of the total waste in some projects. The well-characterized nature of plaster mortars makes them ideal for reuse in new mortar formulations for both modern and historic structures, offering significant environmental benefits.

### 1.1. Aggregate Replacement

The replacement of aggregates in mortars and concretes has been extensively studied in the state-of-the-art literature, particularly due to the potential of incorporating several types of waste materials into their compositions. Table 1 summarizes the analyzed studies along with the key conclusions drawn from each case.

The analysis of the state-of-the-art literature presented in Table 1 reveals the following correlations regarding the replacement of aggregates with waste:

1. Optimal replacement levels—Catarina N. et al. [4] suggest that at a replacement level of 20%, the physical and mechanical properties of mortars remain largely unaffected, whereas Mora-Ortiz et al. [15] demonstrate that up to 60% of natural aggregates can be replaced with recycled fine aggregates without causing substantial changes in the properties of the mortar.
2. Effect of an increased replacement proportion—Nedeljković et al. [5] observed significant decreases in the elastic modulus and compressive strength when 100% of natural aggregates were replaced with recycled ones, suggesting that higher replacement proportions can lead to performance deterioration. Wang et al. [11], Patra et al. [9], and Bao et al. [10] also report that exceeding 30% recycled aggregates results in a drastic reduction in mechanical strength of up to 50%.
3. Apparent density and porosity—Zega et al. [6], Agrela et al. [7], and Zhiming et al. [8] report a correlation between increased porosity and a reduced apparent density of concrete as the proportion of recycled aggregates rises. The negative effects of using recycled aggregates can be offset by CO2 treatment, which improves mechanical properties and reduces water absorption, as suggested by Chinzorigt et al. [12].
4. Deformability and thermal conductivity—Tam V. [14] suggest that variations in aggregate-to-cement and water-to-cement ratios complicate predictions related to shrinkage and deformation, especially when using recycled aggregates. Selvaranjan et al. [13] illustrate that mechanical strengths can be maintained within standard limits and that thermal conductivity can be improved if burnt rice husk ash is used as a substitute for river sand.

In conclusion, these studies indicate that the proportion of recycled waste must be carefully managed to maintain the characteristics and performance of construction materials. While recycling aggregates can promote sustainability and provide viable solutions for waste reduction, the analysis of the results emphasizes the importance of evaluating physical and mechanical characteristics based on the levels of substitute utilized. As in the current paper, 45% of the aggregates are replaced with old plastering mortar.

### 1.2. Cement Replacement

The large-scale study and analysis of replacing cement with various construction wastes has gained considerable attention, particularly due to the advantages of reducing cement usage. Since cement production is a major contributor to greenhouse gas emissions, this approach not only conserves a valuable resource but also offers significant environmental benefits by mitigating carbon emissions. Cement replacement in the state-of-the-art literature is presented in Table 2.

The analysis of the state-of-the-art literature presented in Table 2 reveals the following correlations regarding the replacement of cement with waste:

1. Optimal replacement levels—Miaoyi Deng [22] and Aref A. Abadel et al. [25] reported that a 15% replacement ratio leads to minimal reductions in compressive strength, emphasizing the importance of limiting waste content to maintain mortar quality.
2. Effect of increased replacement proportion—Most studies indicate that excessive waste content leads to performance deterioration, particularly in mechanical properties. Veera Horsakulthai [20] suggested an optimal replacement ratio of 20% for minimal reduction in compressive strength. Similarly, Shujun Li [21] found that both consistency and initial fluidity of mortars decrease as the replacement ratio increases.
3. Performance and hydration process—Wu et al. [16], Patel et al. [17], and Naceri et al. [18] observed a slower hydration process with recycled aggregate powder, fine glass powder, and brick powder, respectively, used as partial replacements for cement at levels of 20–30%. Jeonghyun K. et al. [19] noted that increasing the percentage of recycled concrete waste powder results in lower density and poorer mortar performance, which can also be attributed to the slower hydration process that could contribute to reduced mechanical properties.
4. Mechanical integrity and porosity—Rishath Sabrin et al. [24] and Veera Horsakulthai [20] reported that the decline in compressive strength corresponds to an increase in porosity, indicating that as the waste content rises, both the setting time and consistency are adversely affected. This relationship underscores the importance of managing porosity through controlled waste substitution.

In conclusion, these studies demonstrate that careful management of recycled material proportions is necessary to precisely control the physical and mechanical properties of mortars, ensuring a balance between sustainability goals and material efficacy.

This article proposes a study of these types of mortars, following the analysis of both the specialized literature and the experimental research conducted by the authors of this article. These studies, which are also published in other scientific papers, concluded that the replacement percentages, both for aggregates and for cement, are the ones chosen and described below. Thus, it is proposed to analyze and study the behavior of the compounds in the different studied recipes both at the microscopic level and in terms of the evolution of mechanical strengths over time, with the final goal being the development of a new feasible material for use in construction. This material would make a significant contribution to sustainable development by reusing waste from old plasters.

## 2. Materials and Methods

In our experimental program, all materials utilized conform to the prevailing regulatory standards [26,27,28]. By investigating the reuse of plaster mortar waste, the current paper aims to uncover sustainable practices that can significantly mitigate the environmental impacts of construction and demolition activities, promoting a circular economy within the building sector.

Derived from the renovation efforts of a historic facade in Cluj-Napoca, Romania, the materials used in this experimental study involve the repurposing of waste plaster. This project necessitated the removal of all existing, degraded exterior plaster. The collected plaster waste was carefully extracted directly from the brick support layer of the facade undergoing restoration. Following its extraction, the waste was meticulously separated from other types of construction debris and then categorized into various grades to be effectively used as a substitute for aggregates in plaster mortar formulations.

According to the restoration project documentation of the historic building, the original exterior plastering mortars were composed of cement and lime binder. The sorting and preparation of this waste were conducted within the building materials testing laboratory at the Faculty of Civil Engineering, Technical University of Cluj-Napoca. This process ensured that the waste was properly prepared for experimental use.

### 2.1. Sample Preparation

In current research, 3 types of recipes have been studied, MIX1_REF, MIX3_45_AGG, and MIX2_10_CEM, with the following particularities:

- MIX1_REF—represents the control recipe where no waste was added;
- MIX3_45_AGG—represents the recipe where 45% of the aggregates were replaced with plastering mortar waste;
- MIX2_10_CEM—represents the recipe where 10% of the cement was replaced with old plastering mortar waste.

According to Table 3, the materials used to prepare the samples are the following:

1. Cement—Portland 52.5 R type, Holcim Extradur 52; the cement was kept in the laboratory, respecting all the prescriptions of SR EN 196 [29];
2. Lime—Carmeuse Supercalco M, hydrated powder lime;
3. Aggregates—river sand equally divided into 3 particle sizes—(0–0.5) mm, (0.5–1.00) mm, and (1.00–2.00) mm;
4. Waste—plastering mortars collected from the historic building façade in Cluj-Napoca, Romania, divided into 3 particle sizes—(0–0.5) mm, (0.5–1.00) mm, and (1.00–2.00) mm; the bulk density in loose state was 1066.20 kg/m^3^;
5. Water—potable water in different quantities depending on the sample.

The mortars under examination, comprising both standard plastering mortars and those incorporating plaster waste, were prepared and evaluated in accordance with the norms in force. The process involved dividing the aggregates into equal quantities for particle size as presented above, measuring the required materials (cement, lime, aggregates, and water), and mixing them using an automatic mortar mixer. Following thorough homogenization, the plaster mortar samples were analyzed in both fresh and hardened states by casting them into standardized prisms (40 × 40 × 160 mm).

For optimal compaction, the prisms were placed on a vibrating table and then stored in a humid room with a constant temperature of 20 °C and a relative humidity of 65%-70%. This controlled environment was maintained until the mechanical strength tests were conducted at different curing intervals of 7, 14, and 28 days, as shown in Table 4.

According to Table 4, the analyzed mechanical strengths at the ages of 7, 14 and 28 days are as follows:

- Apparent density—determined for 3 samples at each age by weighing each sample and dividing the obtained mass by the actual prism volume [30];
- Bending strength—determined for 3 samples at each age with the specific apparatus using the standard formula where the force applied to break the sample is multiplied by 1.5 times the distance between the bearings and then divided by the cube of the length of the prism side [30];
- Compressive strength—determined for 3 samples at each age with the specific apparatus using the standard formula where the force applied to break the sample is divided by the area of prism section [30];
- Adhesion to the support layer—determined with the controls of the machine using traction pills bonded to the surface of the mortar, and afterwards the tensile force is applied manually until the sample is removed from the support layer.

### 2.2. Investigation Methods

Mechanical properties were investigated using the automatic machine for loading from bending and the hydraulic press 250 kN Technotest at the ages of 7, 14 and 28 days, which was operated for the compression and flexural strength determinations, respectively. Adhesion to the support layer was performed with a control dynamometer at the age of 28 days for each recipe. [3]

The mineral phases were investigated by X-ray diffraction (XRD) using a Bruker D8 Advance diffractometer produced by Bruker Company (Karlsruhe, Germany) using Cu K_α_ monochrome radiation λ = 1.54056 Å. The investigation range was between 10 and 70 2 theta degrees with a speed of 1 deg./min. Diffraction peak matching was operated with Match 1.0 software produced by Crystal Impact Company (Bonn, Germany) equipped with powder diffraction files in database PDF 2.0.

The correlations of the identified minerals with particles shapes were established through the optical mineralogical microscopy (MOM) effectuated under cross polarized light on a Laboval 2 microscope produced by Zeiss Company (Oberkochen, Germany) equipped with image digital acquisition system Samsung 10 MPx from Samsung Company (Seoul, Republic of Korea).

The samples’ microstructures and fractography were investigated by scanning electron microscopy (SEM) on an Inspect S Microscope (FEI Company, Hillsboro, OR, USA) operated in the low-vacuum mode at an acceleration voltage of 25 kV.

## 3. Results

### 3.1. Mechanical Properties’ Results

#### 3.1.1. Apparent Density

The evaluation is conducted on three prisms, 28 days after the casting of the specimens. Equation (1) presents the calculation formula for the apparent density.

Table 5 shows the average results for apparent density testing at the mentioned intervals of 7, 14 and 28 days.

At 7 days, MIX2_10_CEM exhibits a reduction in value of 3.6%, while MIX3_45_AGG demonstrates a decline of 11.3% compared to the standard MIX1_REF.

At 14 days, the MIX2_10_CEM presents a decrease of 4.4%, and MIX3_45_AGG shows a reduction of 11.15% relative to the standard MIX1_REF.

At 28 days, MIX2_10_CEM displays a value reduction of 3.8%, and the MIX3_45_AGG reveals a decrease of 11.4% when contrasted with the standard MIX1_REF.

The findings demonstrate that mortars containing plaster waste are lighter than those without waste. All results comply with existing regulatory standards. The analysis of the tested mortars indicates that the cement hydration process occurs effectively, and the hardening process is not significantly affected by the inclusion of waste in the proposed mortar formulations at 28 days. This stability is due to the evaporation of water during the curing process.

#### 3.1.2. Bending Strength

To objectively evaluate the performance of the proposed and studied mortars while considering the incorporation of plastering mortar waste from the rehabilitation of historical building facades, the experimental program includes monitoring the physical and mechanical properties of the mortars over periods of 7, 14, and 28 days.

Table 6 illustrates the bending strength values obtained at these intervals for each specific recipe examined.

At 7 days, MIX2_10_CEM shows a reduction in value of 34.6%, while MIX3_45_AGG demonstrates a decline of 25.51% compared to the standard MIX1_REF.

At 14 days, the MIX2_10_CEM presents a decrease of 36.33%, and MIX3_45_AGG shows a reduction of 25.11% relative to the standard MIX1_REF.

At 28 days, MIX2_10_CEM displays a 5.85% increase in value, and the MIX3_45_AGG reveals a decrease of 23.5% compared to the standard MIX1_REF.

Replacing the aggregates and cement with plastering mortar waste resulted in reductions in bending strength values by up to 36% at 7 days and up to 23% at 28 days in comparison to the standard recipe.

#### 3.1.3. Compressive Strength

All the results obtained for the compressive strength are presented in Table 7 at the same intervals as the apparent density and bending strength.

At 7 days, MIX2_10_CEM shows a reduction in value of 35.46%, while MIX3_45_AGG presents a decline of 33.33% compared to the standard MIX1_REF.

At 14 days, the MIX2_10_CEM demonstrates a decrease of 26.68%, and MIX3_45_AGG shows a reduction of 37.34% relative to the standard MIX1_REF.

At 28 days, MIX2_10_CEM reveals a 26.8% decrease in value, and the MIX3_45_AGG displays a decrease of 38.47% compared to the standard MIX1_REF.

The substantial variation between MIX2_10_CEM and MIX3_45_AGG relative to MIX1_REF can be attributed to the increased volume of waste that replaced the aggregates in the mixture.

#### 3.1.4. Adhesion to the Support Layer

Testing of the recipes was conducted at 28 days for all the studied mixtures, and all the results are shown in Table 8.

Compared to MIX1_REF, the value for MIX3_45_AGG shows a 20% decrease whereas MIX2_10_CEM displays a 74.22% increase in value.

In conclusion, the integration of waste into the samples consistently leads to reduced values for the evaluated properties in the case of more than a 10% quantity of waste. This decline is evident especially when it is used as a substitute for aggregates. This suggests that the presence of waste materials, regardless of their specific role, adversely affects the overall performance and structural integrity of the plastering mortars.

### 3.2. Mineralogical Investigations

**Figure 1:**
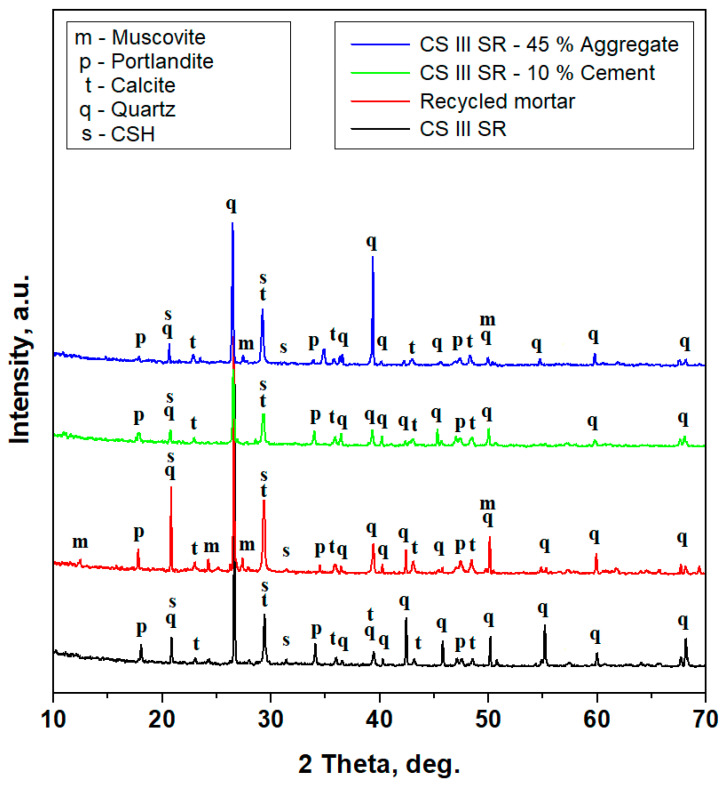
The XRD patterns for the granular material that resulted after the mechanical properties were tested.

The XRD patterns obtained for the granular material that resulted after the mechanical properties were tested are presented in Figure 1. Their general aspects are marked by narrow and intense diffraction peaks, indicating a crystalline structure of the samples, and several less intense peaks having broadened aspects are observed, a fact that indicates the presence of fine fractions in various amounts.

The XRD pattern obtained for the mortar sample having a standard cement recipe (MIX1_REF) is dominated by a very intense and narrow peak belonging to quartz, which is the dominant mineral. There are intense and narrow peaks for calcium silicate hydrate (CSH) and portlandite. Some of the portlandite peaks are less intense and moderately broadened, indicating the presence of fine microstructural grains. Calcite is represented by relatively less intense diffraction peaks, excepting the one at 29.43 degrees that is overlapped with one of the most relevant CSH peaks. The relative intensity reference (RIR) method was applied to establish the weight amount of each mineral in the sample [31,32]. It is based on quantifying the relevant peaks’ relative intensities with the mineral corundum factor. The obtained values are centralized in Table 9.

The recycled mortar sample is dominated by quartz, which has very intense and well-developed peaks with a narrow allure, followed by calcite, which has stronger diffraction peaks that bring extra intensity to the 29.43-degree peak, indicating a significant appreciation of the calcite amount regarding the CSH presence. Portlandite is represented by significant relatively intense and narrow peaks, such as the one at 17.84 degrees, and the other peaks are rather broadened, such as the one at 47.05 degrees. The portlandite peaks’ aspects indicate a significant presence but merely having a finer microscopic fraction than bigger crystallized particles. Muscovite presence is remarkably interesting due to the firmly developed peaks with narrow shapes that indicate the presence of bigger particles, and their small intensities is related to the low amount of muscovite in the recycled mortar sample. The amount of each mineral was established by the RIR method, and the values are centralized in Table 9.

The sample MIX2_10_CEM has 10% of the cement replaced with recycled mortar, a fact that influences the mineral composition of the sample. Quartz is still the dominant mineral due to its most intense and narrow peak at 26.57 degrees, but the CSH peak at 31.39 degrees completely disappears, indicating a significant decrease in the amount of this mineral, a fact that facilitates a relative prevalence of portlandite peaks, which are relatively more intense but still broadened. Calcite peaks are well developed with narrow shapes but less intense, indicating a moderate amount. It would have been expected to find some muscovite as a trace mineral from the recycled mortar, but it does not appear, meaning that it is situated below the detection limit, see Table 9.

The sample MIX3_45_AGG has replaced 45% of the aggregate with recycled mortar. Therefore, the mineral composition is strongly influenced by the significant amount of the replaced material. The most relevant change that occurred in this sample is the detection of muscovite, as evidenced by low-intensity peaks with a narrow shape, indicating the presence of some large muscovite particles. Besides this meaningful change, the quartz dominance is not affected nor is the CSH presence, indicating that cement was under standard prescriptions. Portlandite peaks are also well developed but some of them are broadened due to the fine fraction’s occurrence. Calcite has less intense peaks but with narrow shapes, indicating the presence of some well crystallized particles. The relative intensities of the most relevant peaks allow the proper calculation of each mineral amount, see Table 9.

The aggregate minerals are quartz calcite and traces of muscovite where it occurs, as it is related to the granular material within mortar composition. Hence, the standard recipe is based on lime and Portland cement; their hydration products bring cohesion to the mortar structure by binding the aggregate particles. Therefore, all XRD patterns evidence the presence of portlandite as consequence of the lime hydration and calcium silicate hydrate as a consequence of the hydration of tricalcium silicate (from the cement composition). Both minerals are responsible for the mechanical properties’ achievement.

Mineral distributions within the sample’s microstructure play an important key role in the mortar binding abilities. Mineralogical optical microscopy (MOM) allows the relation between the identified minerals and their distribution among the sample’s microstructures to be followed.

**Figure 2:**
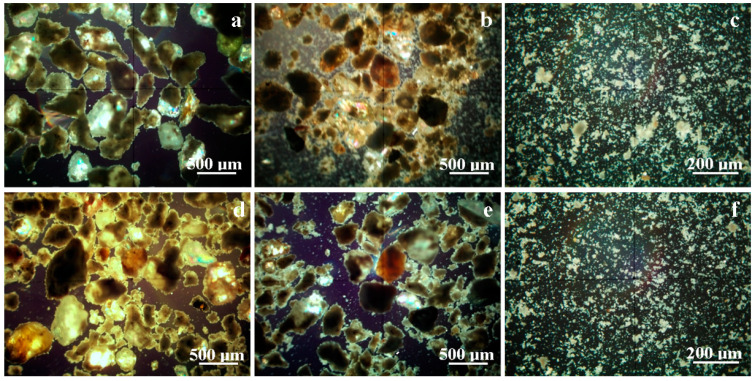
Mineralogical optical microscopy images for the granular material that resulted after the mechanical properties were tested: (**a**) MIX1_REF, (**b**) recycled mortar, (**c**) fine fractions of recycled mortar, (**d**) MIX2_10_CEM—10% of cement replaced by recycled mortar, (**e**) MIX3_45_AGG—45% of aggregates replaced by recycled mortar, and (**f**) fine fractions of MIX3_45_AGG replaced by recycled mortar.

Figure 2a evidences the microstructural aspect of the MIX1_REF sample. The observation field is occupied by large quartz particles featuring the standard green–grey nuance, having a boulder-like shape and sizes ranging from 400 to 600 µm, a fact that is in good agreement with previous observations mentioned in the literature [31,32]. Calcite particles have a general aspect of rounded boulders with brown and yellow to dark brown nuances, depending on their size and position along the optical axis of the microscope, a fact that is in good agreement with the literature [33]. Calcite particle sizes range from 250 to 500 µm, having a good resemblance to the quartz ones, meaning that they appear as natural occurrences from the aggregate sand source. Both quartz and calcite particles are partly covered with a dense crust of the adhesion layer formed by portlandite and the CSH fine admixture, having a light greenish nuance. The literature data reveal a light brown–grey nuance for the portlandite crystals found in cement pastes [34] and pale blue– gray shade in mortar pastes [35] during the MOM investigation. Thus, the portlandite color nuance observed under MOM might vary under the deposit forms as observed by Nijland et al., who evidenced the crust of small portlandite crystals colored in a yellow-greenish shade due to the random positioning of the individual small particles that exhibit a slight overlap [36]. CSH particles within concrete and mortars usually have a yellow nuance during MOM observations [37]. Thus, the portlandite and CSH fine particle admixture has a light greenish nuance observed under MOM. The detailed view of this crust microstructure will be evidenced by the high-magnification SEM imaging.

The recycled mortar sample has a heterogeneous microstructure based on bigger fractions of quartz and calcite particles, as shown in Figure 2b. Quartz particles have boulder-like shapes with sharp edges and a green–gray nuance, while calcite particles have rounded edges and a predominantly brown nuance. The increased amount of calcite particle is sustained by the XRD investigation and shows that the older mortar was prepared with local river sand without special preparation. This fact is sustained by the particle size range that varies from about 10 μm to over 300 μm. The aggregate particles’ source of origin in the alluvial sedimentary deposits explains the presence of larger muscovite particles (predominantly pink nuances, in agreement with the literature data [38]) of about 10–50 μm. The MOM investigation confirms the muscovite amount of 8 wt.% that resulted from the XRD patterns. Figure 2b shows that the bigger aggregate particles are surrounded by a dense mass of fine particles having a heterogeneous constitution featuring yellow–greenish nuances. These fine particles are observed at high magnification in Figure 2c, featuring a yellow–greenish shade caused by the admixture of portlandite and CSH fine particles forming dendritic clusters with relative sizes below 10 μm. The large amount of fine particles of binding agent within the recycled mortar sample indicates its structural alteration that developed over 50 years of exposure to weathering conditions.

Replacing 10% of the cement with recycled mortar in the sample MIX2_10_CEM significantly increases the amount of aggregate with uneven particles. Therefore, many quartz and calcite particles with sizes below the standard range of 400–600 μm occur. The significant amount of recycled mortar fine fractions is merely embedded on the bigger particle surface as a consequence of the mortar hydration and consolidation and turns the standard green–gray nuance to yellow. The replacement amount is too low for muscovite to appear as a distinct phase in this sample. The lack of individual small fractions sustains the system cohesion.

The replacement of aggregate particles with 45% recycled mortar in MIX3_45_AGG sample induces an advanced alteration of the microstructural particles’ distribution due to the presence of smaller fractions of quartz and calcite in the range of 10–400 μm below the standard grain class, as shown in Figure 2e. Several muscovite particles are observed in the size range of 10–50 μm, in good agreement with the XRD observation. The portlandite and CSH fine fractions within the recycled mortar are partly embedded into the newly formed adhesion layer, and some of them were detached under the broken load, forming small micro-sized clusters. Maintaining the standard amount of cement in this sample ensures a normal nuance of the adhesion layer attached onto the aggregate particles. Figure 2f shows the aspect of the fine fractions detached from the MIX3_45_AGG sample during mechanical testing. They have a strong resemblance to the fine fractions occurring in the recycled mortar, indicating a local weakness of the aggregate particles binding during the lime and cement hydration process.

### 3.3. Microstructure and Fractography Assessments

The advanced microstructural aspects were investigated by scanning electron microscopy on the fracture products obtained after mechanical testing. This approach ensures an optimal view of the sample’s microstructure and their failure mode.

**Figure 3:**
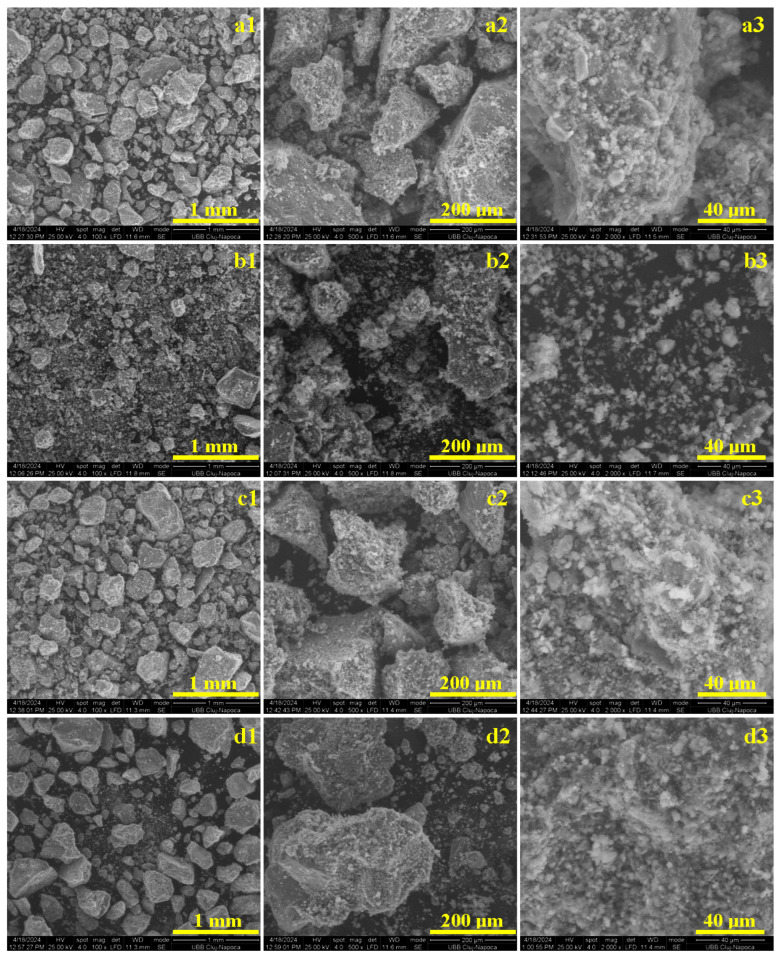
SEM images of the granular material that resulted after the mechanical properties were tested: (**a1**–**a3**) MIX1_REF, (**b1**–**b3**) recycled mortar, (**c1**–**c3**) MIX2_10_CEM—10% of cement replaced by recycled mortar, and (**d1**–**d3**) MIX3_45_AGG—45% of aggregates replaced by recycled mortar. SEM observations were effectuated at several magnifications: (1) 100×, (2) 500×, and (3) 2000×.

The sample with the standard mortar recipe has a granular breaking product in which the bonding between the aggregate particles is disintegrated due to the effort of spatial spreading through the microstructural constituents, as shown in Figure 3a1. The particles’ sizes and shapes are consistent with the MOM observation, revealing predominantly bigger particles in the range of 400–600 µm but some bigger formations that contain few aggregate particles still bonded together also occur. A closer look at the particles within the failure debris in Figure 2 reveals with high accuracy the boulder shape of the quartz aggregate particles with sharp edges, while calcite particles are predominantly rounded (those two particles are situated in the upper left corner of Figure 3a2). All aggregate particles are covered by a dense layer of fine crystals belonging to the adhesion layer that was destroyed by the mechanical solicitation. The microstructural detail in the image in Figure 3a3 was taken at high magnification, revealing the fine particles within the broken adhesion layer. There is a mixture of portlandite plaquettes having a length of about 3–10 µm and width of 1–5 µm, shapes and sizes that are in good agreement with the literature data [39,40]. These are surrounded by fine fractions of CSH particles with sizes in the range of 1–5 µm, which are in good agreement with the literature observations [41,42].

The heterogeneity of the recycled mortar sample is clearly evidenced by the low-magnification image shown in Figure 3b1. Large quartz particles evidence sharp edges, while the calcite particles have predominantly rounded edges. Muscovite particles are slightly rare and predominantly big, such as the tabular particle situated in the right lower corner of Figure 3b1 having a pseudo-hexagonal shape and planar diameter of 700 μm. Its thickness is difficult to appreciate due to its position in the observation field. The adhesion layer is partly attached on the aggregate particles within the recycled mortar sample, as shown in Figure 3b2, featuring significant deposits of portlandite and the CSH mixture of fine particles in the size range of 1–10 μm. Unfortunately, most of the adhesion layer is disintegrated into the component particles, as observed in Figure 3b3. Both portlandite and CSH particles are found in the range of 1–20 μm, having a high clustering tendency, a fact that is in good agreement with the MOM observations.

The breaking of the MIX2_10_CEM sample occurs through the adhesion layer, which fails under progressive solicitation. The aggregate particles become detached from one another, keeping the adhesion layer attached on their surface, as shown in Figure 3c1. The sample is dominated by the large quartz particles from the standard aggregate, and some rounded calcite particles are observed that belong to the added recycled mortar. Figure 3c2 gives a closer look over the broken debris particles evidencing the adhesion layer that remains. Its aspect is rather plucked when the effort dissipated among aggregate particles acts perpendicularly on the adhesion layer, subjecting the component particles to tensile solicitation. Thus, the 10% cement lacking from the standard recipe causes a diminishing of the CSH presence, weakening the adhesion and letting the sample cohesion on the portlandite platelets develop. The higher-magnification images in Figure 3c3 reveal the abundance of portlandite particles in the range of 3–15 μm, some of which are horizontally positioned regarding the quartz particle surface while the others are displaced on their side as consequence of tensile plucking. The CSH particles appear randomly disposed as fine dots of about 1–5 μm and cannot play their key role due to their reduced number. The microstructural details indicate an inter-granular failure of the adhesion layer.

Replacing 45% of aggregate particles with recycled mortar in sample MIX3_45_AGG does not affect the active ingredients (e.g., lime and cement) in the standard recipe. It certainly influences the aggregate grain size distribution. The presence of a significant amount of fine fractions from the old adhesion layer interacts with lime and cement during the hydration process and interferes with the bonding crystal development. The overall aspect of the fractured debris particles in Figure 3d1 reveal quartz and calcite aggregate particles that are well covered with the adhesion layer that remains, but detachment of the fine fraction occurs. It indicates that the finer fractions within the recycled mortar do not participate in sample cohesion and easily can be unbonded from the adhesion layer. The aspect is more evident in Figure 3d2, where the adhesion layer is partly unbonded from the quartz particle surface, which has significant areas without adhesion layer particles and can be observed as individual fine fractions on the right side of the figure. The adhesion layer that still adheres to the quartz particle surface is further observed at high magnification in Figure 3d3. The newly formed portlandite and CSH particles tends to bring bonding bridges to the quartz surface, but several at the center of the observation field appear as a clogging cluster that embeds older portlandite particles from the recycled mortar that reduces the binding ability of the new formed crystals.

## 4. Discussion

Both the mechanical properties and mineralogical analyses of the samples indicate that incorporating old plastering waste tends to degrade the macroscopic and microscopic characteristics of the material. This observation aligns with findings from the state-of-the-art literature, where replacing either cement or aggregates with waste at higher percentages similarly resulted in reduced performance.

Table 10 illustrates the differences between the results obtained in the current study and those reported in the literature when aggregates were substituted with waste in proportions ranging from 40% to 50%.

According to Table 11, the standard recipe MIX1_REF yielded the best results in terms of mechanical strength. This finding is further corroborated by mineralogical and scanning electron microscopy (SEM) analyses. Microstructural analyses indicate that quartz is the predominant mineral across all the recipes examined, accompanied by varying proportions of calcite, portlandite, calcium silicate hydrate (CSH), and muscovite, depending on the specific recipe.

A common observation in all the recipes, including mortar waste, is that quartz particles exhibit sharp edges, while calcite particles have rounded edges. The old plaster waste used in these recipes is characterized by aggregates ranging from 10 to 300 μm (river aggregate), portlandite and CSH particles ranging from 1 to 20 μm, and a relatively high proportion of muscovite particles (8%) with large, tabular dimensions of up to 700 μm. This composition supports the idea of reduced mechanical strength when such waste is incorporated into the analyzed recipes. The introduction of waste into mortar or concrete recipes has been shown to decrease mechanical strength, as also noted in the literature in the article by Saitis et al. “Recycling Plaster Waste as a Substitute for Aggregates in Obtaining Plastering Mortars” [3], where a comprehensive analysis of mechanical strengths based on the percentage of aggregates replaced with waste was conducted.

For the standard recipe MIX1_REF, which contains no old mortar waste, the quartz particles range from 400 to 600 μm and the calcite particles from 250 to 500 μm. In this scenario, the proportion of binder (portlandite + CSH) present in the composition, being the highest among the studied recipes (41%), results in superior mechanical strengths (both flexural and compressive). Adhesion to the substrate is directly influenced by the quantity of quartz and portlandite (68%) in the studied recipes. Accordingly, in Table 11, the mineralogical analysis and mechanical strengths indicate that the obtained value of 0.025 N/mm^2^ lies within the range of the other two values obtained.

In the recipe MIX2_10_CEM, the quartz and calcite particles are smaller than 400–600 μm, the portlandite particles range from 3 to 15 μm, and the CSH particles range from 1 to 5 μm. During the hydration process of the mortar, the fine particles are incorporated on the surface of the larger particles. However, due to the reduced amount of binder (cement), there is a decrease in the CSH content, leading to reduced adhesion between the mortar components and resulting in disordered bonding. Additionally, there is an increase in the uneven distribution of aggregates, resulting in lower mechanical strengths compared to the standard recipe MIX1_REF. The obtained value for adhesion to the substrate is higher than in the other two recipes, which is attributed to the higher content of portlandite + quartz in the mortar composition (78%).

In the recipe MIX3_45_AGG, the quartz and calcite particles range from 10 to 400 μm, presenting smaller fractions compared to the other recipes, which significantly alters the distribution of microstructural particles. Despite this, the active components of the mortar (lime and cement) are not significantly affected by the presence of waste, as indicated by the calcite and quartz particles being well-encapsulated by the binder. The bonds formed between particles during the hydration process of lime and cement (which are weaker) resemble those observed in the analyzed old mortar due to the high proportion of waste replacing the aggregates. Furthermore, the fine fractions appear detached, as they interfere with the crystal adhesion formation process, and the portlandite particles are directly incorporated from the recycled mortar, reducing the capacity to form bonds between the new crystals. Consequently, the recorded mechanical strengths are the lowest, including adhesion to the substrate, with portlandite and quartz being present in a proportion of 59%, which is less than in the other cases.

## 5. Conclusions

Taking into consideration the macroscopic and microscopic analyses of the studied recipes, the following observations can be made:

1. Apparent density—From 7 to 28 days, the samples become lighter due to water evaporation. The apparent density decreases with increasing waste content, making the samples lighter. This is supported by the mineralogical analysis, showing 25% calcite (highest), 36% quartz (lowest), and 8% muscovite, a lightweight compound that weakens the mortars.
2. Flexural strength—Adding waste reduces flexural strength by about 24% after 28 days for the MIX1_REF 45% aggregate recipe. However, the MIX1_REF 10% cement recipe shows comparable results to the standard. The microscopic analysis reveals that portlandite from the waste does not fully bind, weakening mechanical strength.
3. Compressive strength—Waste reduces compressive strength by 38% for MIX1_REF 45% aggregates and 27% for MIX1_REF 10% cement at 28 days, compared to the standard. This is due to the uneven particle distribution, weakening adhesion during hydration and disrupting crystal formation.
4. Adhesion to the support layer—Waste negatively impacts adhesion when exceeding a 10% aggregate content, with a lower portlandite+quartz proportion. In the MIX1_REF 10% cement recipe, waste improves adhesion, yielding values nearly four times higher than the standard. Higher waste levels, however, increase the muscovite content, weakening adhesion.

### Innovative Contributions and Environmental Impacts

The current research paper builds on the known sequence of curing and the impact of waste inclusion but provides new, specific quantitative data and mineralogical insights. It offers a detailed understanding of how the waste composition (specifically calcite, quartz, and muscovite contents) affects the density, strength, and adhesion to the support layer as presented below.

1. Apparent density—The lighter weight of mortars with a greater waste content is linked to specific mineralogical properties—such as the high calcite and low quartz contents—coupled with the presence of muscovite, a lightweight compound that negatively affects mortar properties;
2. Flexural strength—The current study explains the reduction in value through a microscopic analysis, identifying that portlandite particles from the waste do not fully engage in binder formation, which provides deeper insights into the strength reduction mechanism;
3. Compressive strength—The reduction is attributed not only to particle non-uniformity but also to the disruption of adhesion bonds during hydration; a disorder in the formation of binding crystals is highlighted by adding specificity to the mechanism behind the strength reduction that goes beyond the usual explanations of mechanical weakening;
4. Adhesion to the support layer—In the current research, the differentiated behavior of the samples was observed based on the mineral composition and the fact that the presence of 78% quartz + portlandite enhances adhesion.

Regarding environmental and sustainability considerations, this study demonstrates that incorporating waste materials in mortars can reduce the reliance on virgin resources, leading to lower carbon emissions and energy consumption. The findings highlight the possibility of balancing mechanical performance with environmental benefits. By reusing waste aggregates and cement substitutes, the construction industry can make strides toward more sustainable practices, reducing landfill waste, and promoting material circularity. The fact that waste can be effectively used at up to 10–15% in mortars while still offering adequate performance is key for reducing the overall environmental impacts of cement production.

## Figures and Tables

**Table 1:** Aggregate replacement in the state-of-the-art literature.

Reference/Studied Materials	Waste Used to Replace Siliceous Aggregates	Replacement Ratio [%]	Compressive Strength 28 Days [N/mm^2^]	Bending Strength 28 Days [N/mm^2^]	Conclusions/Observations
Catarina N. et al. [4]/cement mortars	recycled concrete aggregates	20%	5.34	1.86	- Physical and mechanical properties remain largely unaffected at 20% replacement. - A slight reduction in adhesion to the support layer is observed. - Water absorption decreases by up to 25%.
		50%	5.15	1.63	
		100%	7.38	2.15	
Nedeljkovic et al. [5]/concrete	fine recycled aggregates	15%	40.60	3.30	Replacement of fine natural aggregates with 100% fine recycled aggregates led to the following results: - A 17% decrease in the elastic modulus; - The compressive strength decreased by 50%; - The tensile strength dropped by up to 33% compared to standard concretes; - These reductions are attributed to the increased water–cement ratio required to achieve the workability of standard concrete made with natural sand; - A significant increase in creep; - The incomplete water absorption by the dry fine aggregate increases the need for a larger amount of water to achieve the desired consistency.
		30%	42.00	3.20	
		45%	41.70	3.20	
		60%	41.00	3.10	
		100%	40.10	3.00	
Zega et al. [6]/concrete	fine recycled aggregates	20%	42.70	4.4	As the amount of waste used as an aggregate substitute in the concrete mix increases the following can be observed: - Increased porosity; - A reduction in the apparent density of the concrete due to the lower density of the recycled waste; - The compressive strengths of concretes with 20% and 30% recycled fine aggregate are nearly identical to those of concrete made entirely with natural fine aggregates; - Water absorption increases by up to 15%; - All the reductions comply with the norms in force.
		30%	41.40	4.00	
Agrela et al. [7]/concrete	recycled concrete aggregates	0–100%	-	-	- A strength loss of up to 6% can be anticipated for concretes with a compressive strength below 40 N/mm^2^ when substituting up to 50% of the material. - Concerning water absorption, the impact of the used waste is negligible when only lesser amounts of recycled coarse concrete aggregate (20–30%) are used. - Regarding flexural strength, the differences between recycled and conventional concretes are minimal showing a 10% reduction when recycled concretes were used. - The same reduction in mechanical properties was also observed by Zhiming et al. [8].
Patra et al. [9]/concrete	recycled coarse aggregates	20%	28.00	2.35	- Reductions in compressive and structural strengths are not significant until 30% replacements. - Poor adherence between recycled materials is observed. - The weak adhesion between recycled aggregates and excessive water absorption of recycled materials determines the loss in mechanical properties. - Water absorption increases as waste is added.
		30%	26.50	2.25	
		40%	24.00	1.95	
		50%	23.00	1.80	
		60%	22.00	1.70	
Bao et al. [10]/concrete	recycled concrete aggregates	30%	40.00	-	- The compressive strength of the specimens with a 100% replacement ratio decreases by approximately 10.50%. - Water absorption in the studied samples increases with higher waste content, due to greater porosity and larger pores in the recycled aggregates. - Microcracks from processing also weaken absorption resistance, making the concrete more prone to capillary action.
		50%	39.00	-	
		100%	37.00	-	
Wang et al. [11]/concrete, comprehensive review	recycled coarse aggregates	-	-	-	- Replacing natural aggregates with recycled aggregates from construction and demolition waste in recycled aggregate concrete can yield significant environmental and economic advantages. - It is estimated that using recycled coarse aggregates instead of natural aggregates in concrete can reduce material costs by 10% to 20%. - Recycling coarse aggregates can lower greenhouse gas emissions by 65% and decrease non-renewable energy consumption by 58% compared to natural aggregate concrete. - The mechanical properties, including compression, tensile, and bending strengths, are typically lower than those of natural aggregate concrete, which restricts its use in structural applications.
Chinzorigt et al. [12]/concrete	recycled aggregates	10%	49.30	9.50	- The reduction in mechanical strength was less pronounced in concrete with treated recycled aggregates compared to those without treatment. - An increase in density was observed due to the reduced water absorption by recycled coarse aggregates. - Shrinkage performance remained largely unchanged.
		30%	40.80	8.40 7.40	
		60%	40.80		
Selvaranjan et al. [13]/cement mortars	open-burnt rice husk ash	10–50%	-	-	- A reduction in the thermal conductivity of mortars containing ash from uncontrolled combustion by up to 62% is specifically observed with a 30% displacement of sand. - A reduction in the thermal conductivity of mortars containing ash from controlled combustion by up to 18% is observed. - Adding ash to mortars enhances their thermal performance. - Regarding environmental impacts, CO_2_ emissions from the mortars decreases by approximately 14.7% after replacing 30% of river sand with rice husk ash.
Tam V. [14]/concrete	recycled aggregates	30%	52.40	-	- The maximum drying shrinkage observed is approximately 0.12108% in the sample that has a 100% replacement of recycled aggregates (c-RA), an aggregate-to-cement ratio of 6, and a water-to-cement ratio of 0.45. - Drying shrinkage and creep behavior are increased by increasing the replacement ratios. - The reversibility of drying shrinkage and creep behavior diminishes with higher replacement ratios after the rewetting and unloading of the samples.
		50%	50.00	-	
		70%	46.80	-	
		100%	41.50	-	
Mora-Ortiz et al. [15]/mortars	recycled fine aggregates derived from mortars	20%	4.80	-	- Bulk densities and the compressive and adhesive strengths of the mortars decrease with the rate of aggregate replacement. - Water absorption and the air content increase as the content of recycled aggregates increases because it is necessary to add more water to the mixture to achieve the projected consistency. - A commercial plasticizer can improve mortar’s properties. - The findings demonstrate that incorporating recycled aggregates in place of natural ones is viable up to a 60% replacement ratio without causing substantial alterations in the performance or key properties of the mortars when compared to a control sample made with conventional aggregate.
		40%	4.10	-	
		60%	3.50	-	
		100%	3.00	-	

**Table 2:** Cement replacement in the state-of-the-art literature.

Reference/Studied Materials	Waste Used to Replace Cement	Replacement Ratio [%]	Compressive Strength at 28 Days [N/mm^2^]	Bending Strength at 28 Days [N/mm^2^]	Conclusions/Observations
Wu et al. [16]/concrete	recycled fine powder from concrete waste	30%	-	-	- Increase in pore structure; - Reduction in hydration products; - Decrease in permeability and mechanical strengths; - Mechanical strengths could be improved by 35.9% and 38.1% by adding 10% silica fume or 10% metakaolin, respectively; - Recycled fine powder (RFP) derived from various concrete wastes had irregular microstructures and included hydrated components, quartz, or calcite; - Incorporating RFP led to a reduction in the generation of new hydration products.
Patel et al. [17]/cement mortar	fine glass powder with a decrease in particle size of 12 µ	0–20%	-	-	- Glass particles smaller than 75 µm, used to replace 10–25% of Portland cement, showed promising results in producing blended mortars and concrete; - The addition of glass powder as a partial replacement for traditional OPC enhanced several properties of the final product; - The early inclusion of glass powder in cement effectively filled voids in the hydrated cement matrix, resulting in a denser and thicker microstructure in the blended cement–glass powder composition.
Naceri et al. [18]/cement mortar	brick waste powder	5%	45.00	7.40	- Glass particles smaller than 75 µm, used to replace 10–25% of Portland cement, showed promising results in producing blended mortars and concrete; - The mechanical strength of mortars decreased with higher artificial pozzolan content at 7 and 28 days but improved at 90 days for mixes with up to 10% waste brick; - Waste brick reduced the cement grinding time and cutting energy use, and lowered the cement density; - A higher waste content shortened setting times due to increased water absorption, speeding up chemical reactions; - SEM at 90 days showed portlandite crystals around voids, and laser granulometry revealed particles between 0.05 and 878 µm.
		10%	38.75	7.25	
		15%	36.25	7.15	
		20%	35.00	6.95	
Jeonghyun K. et al. [19]/cement mortar	recycled concrete waste powder	10%	32.27	7.75	- Poorer performance of the mortars was produced because the increase in the amount of waste used to replace cement led to a lower powder density; - As the amount of waste used increased, the consistency and mechanical strength decreased; - The negative influence of the waste on the mixtures could be mitigated by reducing the cement replacement ratio.
		20%	27.41	7.11	
		30%	22.90	6.71	
Veera Horsakulthai [20]/self-compacting cement mortar	recycled concrete waste powder	10%	77.10	-	- The compressive strength of self-compacting mortar containing recycled concrete powder decreased as the waste content increased. - Incorporating recycled concrete powder also reduced electrical resistivity, with a more significant drop as the waste percentage increases. At 28 days, resistivity decreased by 18.1%, 37.7%, and 43.6% for 20%, 40%, and 60% waste content. - Porosity increased with a higher waste content, rising by 6.3%, 16.5%, and 26.6% at 28 days for the same replacement ratios. - Water absorption also increased significantly with the waste content, by 34.4%, 70%, and 149.3% at 28 days for 20%, 40%, and 60% waste.
		20%	56.60	-	
		30%	37.90	-	
Shujun Li [21]/cement mortar	concrete waste powder	30%	36.00	6.3	- The initial fluidity was reduced due to wastes with a high specific surface area and porosity, which led to a notable loss in consistency; - The pozzolanic reaction of the waste was minimal, even at later stages, with a maximum activity index of 77% at 90 days; - A harmful interfacial zone between the particles and cement paste was observed in mortars containing concrete powder, causing a deterioration in mechanical properties and durability; - Reduced shrinkage and improved freeze-thaw resistance were observed, with these properties gradually enhancing as the powder’s fineness increased.
Miaoyi Deng [22]/cement mortars	recycled concrete waste powder	5%	22.50	3.40	- Initially, the strength of the cement mortars increased, but then decreased as the waste content grew; - When the percentage of cement replaced by waste was less than 15%, the maximum increase in compressive strength at 3 days was 19.08%, while the maximum decrease in compressive strength at 28 days was 2.39%.
		15%	22.00	3.80	
		25%	18.90	2.70	
Jan Pizon [23]/cement mortars	recycled foam concrete waste	25%	29.80	-	- An acceptable reduction in mechanical properties was observed while improving thermal performance, making it particularly suitable for plasters and mortars; - A decreased thermomechanical index, defined as the ratio between compressive strength and thermal conductivity, was observed; - Density, water absorption, and compressive strength were not significantly affected when cement was substituted with cellular concrete waste (granule sizes of 0.063 mm and 0.125 mm); - Linear correlations between density and thermal conductivity, as well as compressive strength and thermal conductivity, remained within acceptable limits for both substitution scenarios.
		50%	15.40	-	
Rishath Sabrin et al. [24]/cement mortars	recycled concrete dust waste	5%	43.00	6.60	- A reduced consistency and setting time of cement pastes were observed, with values declining as the replacement levels increased; - The consistency for all mixtures ranged from 25% to 27.5%; - Workability decreased by up to 49.8% as the proportion of cement replaced with waste increased; - Compressive strength increased by about 9% up to a 5% replacement, while at elevated temperatures, it dropped by 82% compared to room temperature; - Flexural strength also improved, showing a 9.7% increase up to a 10% replacement; - Water absorption was increased by about 7%, while higher replacement rates increased absorption by up to 36%.
		10%	37.00	6.20	
		15%	29.00	5.80	
		20%	27.00	5.60	
Aref A. Abadel et al. [25]/cement-lime mortars	plaster mortar waste powder	10%	28.50	6.10	- A slight improvements in fresh mortar fluidity was observed with replacement ratios below 10% and above 20%, while a 15% content reduced fluidity by 7%; - Compressive strength decreased with waste addition, and the lowest reduction (17.47%) occurred with a 15% waste mixture compared to the reference mortar; - Flexural strength remained largely unaffected up to 15% replacement but declined at higher levels; - Ultrasonic wave velocity, density, and the dynamic modulus of elasticity decreased with waste inclusion, with the smallest drops observed at 15%; - Water absorption remained comparable to the reference mortar up to 15% replacement; - A microscopic analysis indicated a denser and more homogeneous structure with 15% waste.
		15%	28.40	6.30	
		20%	25.60	5.10	
		25%	23.50	4.90	
		30%	20.40	4.30	

**Table 3:** Materials and recipes. Quantities are listed for 1 m^3^.

Type of Recipe	Cement [kg/m^3^]	Lime [kg/m^3^]	Aggregates [kg/m^3^]	Waste–Plastering Mortar [kg/m^3^]	Water [L/m^3^]	W/b Proportion
MIX1_REF	275	110	1450	0	311	0.80
MIX2_10_CEM	247.5	110	1450	27.5	337.5	0.99
MIX3_45_AGG	275	110	797.5	652.5	381	0.94

**Table 4:** Mechanical strength tests performed at different intervals.

Type of Mechanical Strength	Type of Recipe	Testing Intervals [Days]
Apparent density	MIX1_REF MIX2_10_CEM MIX3_45_AGG	7, 14, 28
Compressive strength		
Bending strength		
Adhesion to the support layer		

**Table 5:** Apparent density [ρ_a_] at different intervals.

Testing Intervals [Days]	Type of Recipe	Calculation Equation [kg/m^3^] [30]		Results [kg/m^3^]	STDEV
7	MIX1_REF	ρ_a_ = (m_m_ − m_v_)/V × 1000 [kg/m^3^]	(1)	2210	24.51
	MIX2_10_CEM			2134	3.79
	MIX3_45_AGG			1960	46.45
14	MIX1_REF			2169	80.22
	MIX2_10_CEM			2072	71.35
	MIX3_45_AGG			1927	5.65
28	MIX1_REF			2044	10.95
	MIX2_10_CEM			1966	20.05
	MIX3_45_AGG			1811	39.69

**Table 6:** Bending strength [f_yd_] at different intervals.

Testing Intervals [Days]	Type of Recipe	Results [N/mm^2^]	STDEV
7	MIX1_REF	3.41	0.32
	MIX2_10_CEM	2.23	0.16
	MIX3_45_AGG	2.54	0.71
14	MIX1_REF	3.66	0.40
	MIX2_10_CEM	2.33	0.20
	MIX3_45_AGG	2.74	0.77
28	MIX1_REF	4.66	0.28
	MIX2_10_CEM	4.61	0.54
	MIX3_45_AGG	3.32	1.52

**Table 7:** Compressive strength [f_ck_] at different intervals.

Testing Intervals [Days]	Type of Recipe	Results [N/mm^2^]	STDEV
7	MIX1_REF	12.69	1.06
	MIX2_10_CEM	8.19	0.63
	MIX3_45_AGG	8.46	0.71
14	MIX1_REF	16.04	0.82
	MIX2_10_CEM	11.76	0.46
	MIX3_45_AGG	10.05	0.77
28	MIX1_REF	21.60	1.38
	MIX2_10_CEM	15.81	2.61
	MIX3_45_AGG	13.29	1.52

**Table 8:** Adhesion to the support layer.

Testing Interval [Days]	Type of Recipe	Results [N/mm^2^]	STDEV
28	MIX1_REF	0.25	0.19
	MIX2_10_CEM	0.97	0.063
	MIX3_45_AGG	0.20	0.0007

**Table 9:** Mineral amounts determined by the RIR method.

Minerals	Amount, wt. %				
	Quartz	Calcite	Portlandite	CSH	Muscovite
Chemical formula	SiO_2_	CaCO_3_	Ca(OH)_2_	CaH_2_O_4_Si	KAl_2_(AlSi_3_O_10_) (F, OH)_2_
MIX1_REF	49	10	19	22	-
Recycled mortar	36	25	18	13	8
MIX2_10_CEM	56	13	22	9	-
MIX3_45_AGG	43	18	16	17	6

**Table 10:** Conclusions from the state-of-the-art literature vs. the current paper’s results.

Reference	Replacement Ratio [%]	Compressive Strength at 28 Days [N/mm^2^]	Bending Strength at 28 Days [N/mm^2^]	Conclusions/Observations The State-of-the-Art Literature vs. Current Paper’s Results
**AGGREGATE REPLACEMENT**				
Current paper’s results	45%	13.29	3.32	Compressive strength - Regarding compressive strength, when 45% of the aggregates were replaced with waste, Nedeljković et al. [5] reported values at 28 days that were three times higher than those obtained in this study; - For 40% aggregate replacement, the results from this paper were 1.8 times lower than those reported by Patra et al. [9], but 3.2 times higher than those obtained by Mora-Ortiz et al. [15]; - For 50% aggregate replacement, all the results from the literature ([8,9,10]) exceeded the values observed in this study, except for those reported by Catarina et al. [4], which were 2.5 times lower. 2. Bending strength - Nedeljković et al. [5] reported values at 28 days that were three times higher than those observed in this study; - For 40% aggregate replacement, the results in this paper were 1.8 times higher than those reported by Patra et al. [9]; - For 50% aggregate replacement, the results reported by Catarina et al. [4] and Patra et al. [9] were half the values obtained in this study, while Zhiming et al. [8] reported values that were 2.3 times higher.
Catarina N. et al. [4]	50%	5.15	1.63	
Nedeljković et al. [5]	45%	41.70	3.20	
Patra et al. [9]	40%	24.00	1.95	
	50%	23.00	1.80	
Bao et al. [10]	50%	39.00	-	
Mora-Ortiz et al. [15]	40%	4.10	-	
**CEMENT REPLACEMENT**				
Current paper’s results	10%	15.81	4.61	Compressive strength - Rishath et al. [24], Aref et al. [25], and Jeonghyun et al. [19] reported 28-day values that were 2.3, 1.8, and 2 times higher, respectively, than those in this study when 10% of the cement was replaced with waste; - The results documented by Rishath et al. [24] for a 5% cement replacement were 2.7 times lower than the results from this study; - For a 15% cement replacement, Miaoyi et al. [22] obtained values that were 1.4 times higher than those observed in the current paper. 2. Bending strength - Rishath et al. [24], Aref et al. [25], and Jeonghyun et al. [19] reported 28-day values that were 1.34, 1.32, and 1.7 times greater, respectively, compared to those in this study when 10% of the cement was replaced with waste; - For a 5% cement replacement, the values documented by Rishath et al. [24] were 1.4 times higher; - For 5% and 15% cement replacement, Miaoyi et al. [22] observed results that were 1.35 and 1.2 times lower, respectively; - For 15% cement replacement, Rishath et al. [24] and Aref et al. [25] reported values that were over 25% higher compared to the results obtained in this study for 10% cement replacement.
Jeonghyun K. et al. [19]	10%	32.27	7.75	
Miaoyi Deng [22]	15%	22.00	3.80	
Rishath Sabrin et al. [24]	10%	37.00	6.20	
	15%	29.00	5.80	
Aref A. Abadel et al. [25]	10%	28.50	6.10	
	15%	29.40	6.30	

**Table 11:** Mineralogical analysis and mechanical strengths.

Type of Recipe	Minerals, Chemical Formula, and Amount, wt. %	Mechanical Strengths [28 Days]		
	Quartz + Calcite + Portlandite + CSH + Muscovite (SiO_2_ + CaCO_3_ + Ca(OH)_2_ + CaH_2_O_4_Si + + KAl_2_(AlSi_3_O_10_) (F, OH)_2_)	Bending Strength [N/mm^2^]	Compressive Strength [N/mm^2^]	Adhesion to the Support Layer [N/mm^2^]
MIX1_REF	49 + 10 + 19 + 22 + 0	4.66	21.6	0.025
Recycled mortar	36 + 25 + 18 + 13 + 8	-	-	-
MIX2_10_CEM	56 + 13 + 22 + 9 + 0	4.61	15.81	0.097
MIX3_45_AGG	43 + 18 + 16 + 17 + 6	3.32	13.29	0.02

## Data Availability

The original contributions presented in the study are included in the article.
